# Hsp72 Is a Novel Biomarker to Predict Acute Kidney Injury in Critically Ill Patients

**DOI:** 10.1371/journal.pone.0109407

**Published:** 2014-10-14

**Authors:** Luis E. Morales-Buenrostro, Omar I. Salas-Nolasco, Jonatan Barrera-Chimal, Gustavo Casas-Aparicio, Sergio Irizar-Santana, Rosalba Pérez-Villalva, Norma A. Bobadilla

**Affiliations:** 1 Department of Nephrology Nefrología y Metabolismo Mineral, Instituto Nacional de Ciencias Médicas y Nutrición Salvador Zubirán, México City, México; 2 Unidad de Fisiología Molecular, Instituto de Investigaciones Biomédicas, Universidad Nacional Autónoma de México, México City, México; University of São Paulo School of Medicine, Brazil

## Abstract

**Background and Objectives:**

Acute kidney injury (AKI) complicates the course of disease in critically ill patients. Efforts to change its clinical course have failed because of the fail in the early detection. This study was designed to assess whether heat shock protein (Hsp72) is an early and sensitive biomarker of acute kidney injury (AKI) compared with kidney injury molecule (Kim-1), neutrophil gelatinase-associated lipocalin (NGAL), and interleukin-18 (IL-18) biomarkers.

**Methods:**

A total of 56 critically ill patients fulfilled the inclusion criteria. From these patients, 17 developed AKI and 20 were selected as controls. In AKI patients, Kim-1, IL-18, NGAL, and Hsp72 were measured from 3 days before and until 2 days after the AKI diagnosis and in no-AKI patients at 1, 5 and 10 days after admission. Biomarker sensitivity and specificity were determined. To validate the results obtained with ROC curves for Hsp72, a new set of critically ill patients was included, 10 with AKI and 12 with no-AKI patients.

**Results:**

Urinary Hsp72 levels rose since 3 days before the AKI diagnosis in critically ill patients; this early increase was not seen with any other tested biomarkers. Kim-1, IL-18, NGAL, and Hsp72 significantly increased from 2 days before AKI and remained elevated during the AKI diagnosis. The best sensitivity/specificity was observed in Kim-1 and Hsp72: 83/95% and 100/90%, respectively, whereas 1 day before the AKI diagnosis, the values were 100/100% and 100/90%, respectively. The sensibility, specificity and accuracy in the validation test for Hsp72 were 100%, 83.3% and 90.9%, respectively.

**Conclusions:**

The biomarker Hsp72 is enough sensitive and specific to predict AKI in critically ill patients up to 3 days before the diagnosis.

## Introduction

Acute kidney injury (AKI) remains a common syndrome in hospitalized patients in the intensive care unit (ICU) and has consistently been associated with increased morbidity and mortality [1]–[3]. The major causes of AKI are ischemic and nephrotoxic injuries [4]. Nearly 15% of hospitalized patients are at risk of developing AKI; however, the incidence increases up to 40–60% in patients admitted to the intensive care unit [5]–[8]. During AKI, many alterations occur at the cellular and molecular level that finally leads to organ dysfunction. Moreover, there is accumulating evidence which supports that patients who survive an AKI episode have a higher risk of developing chronic kidney disease (CKD) in the following years [9]–[12], including the patients with a complete renal function recovery [13].

Advances in reducing this complication have long been delayed by the lack of early and sensitive biomarkers [14]. In spite of creatinine limitations, the current acute kidney injury network (AKIN) and Risk, Injury, Failure, Loss of kidney function, and End-stage kidney disease (RIFLE) classifications for diagnosing AKI are based on the elevation of serum creatinine or urine output reduction [14]–[16]. Over the past few years, many studies have focused on the development of accurate biomarkers for AKI because early initiation of treatment could improve the prognosis for patients with AKI [17]. Therefore, the development of sensitive renal biomarkers is crucial for the identification of new therapeutic strategies for AKI; such biomarkers will facilitate early treatment, injury stratification and monitoring the course of the disease. With the use of the innovative genomic and proteomic tools, several molecules that are up-regulated during AKI in experimental models and humans have been identified and proposed as biomarkers. Among the most commonly used are: neutrophil gelatinase-associated lipocalin (NGAL), which is almost undetectable in normal epithelial renal cells and its expression is induced during AKI [18], [19]; kidney injury molecule type 1 (Kim-1), which is induced on the surface of proximal tubule cells during ischemic or nephrotoxic injury [20]–[22]; and interleukin 18 (IL-18), which is over-expressed in patients diagnosed with AKI [23], [24]. Recently, we showed that heat shock protein 72 (Hsp72) is an early and sensitive biomarker for AKI in rats and humans. Moreover, this novel biomarker was suitable for stratifying different degrees of tubular injury and recovery, and for monitoring a renoprotective intervention in an experimental rat model of AKI [25], [26]. Because in our previous study, we showed that Hsp72 could predict AKI, we designed a study for diagnostic test to evaluate the sensitivity, specificity, and predictive values of Hsp72 compared with other conventional biomarkers in the early detection of AKI in critically ill patients.

## Materials and Methods

This is a diagnostic test study that was performed in accordance with national and international guidelines and regulations, and was approved by Ethics Committee, Instituto Nacional de Ciencias Médicas y Nutrición Salvador Zubiran, México (No. 166) and it was adhered to Declaration of Helsinki. All subjects or designed surrogate (when ICU patients could not sign) signed the informed consent form.

### Inclusion of critically ill ICU patients

A diagnostic test study was performed with only critically ill patients who were recruited during three months in the intensive care unit from our institution and who exhibited two or more organ failures. One of the organ failures had to be respiratory with mechanical ventilation, a glomerular filtration rate (GFR) estimated by the Modification of Diet in Renal Disease (MDRD) Study equation >60 ml/min/1.73 m^2^, and with no AKI at the moment of enrollment.

An aliquot of daily fresh urine sample was collected at 7 o’clock in the morning from all critically ill patients hospitalized in the ICU and stored at −80°C until the biomarkers were measured. Serum creatinine and urinary volume were monitored daily. Accordingly with our gold standard for this study (AKIN criteria), AKI was defined when urinary output was <0.5 ml/kg/h during a 6-h period or when the serum creatinine increased more than 0.3 mg/dl or 1.5- to 2-fold increase from the baseline value.

Urinary Kim-1, IL-18, NGAL, and Hsp72 levels were measured by ELISA technique from 17 patients diagnosed with AKI, from 3 days before until 2 days after the development of AKI. From the daily samples collected from all patients who did not develop AKI, we randomly selected 20 patients to be included in the study. The biomarkers were analyzed only in urine samples from days 1, 5 and 10 after ICU admission. These sampling days were chosen to ensure that the biomarkers levels were representative of all hospitalization period in no AKI patients and supported by the fact that the release of these biomarkers in the urine did not occur so fast. In addition, the mean value of the 3 different urine samples of each biomarker was used to compare the values obtained in patients diagnosed with AKI and for the Receiver Operating Characteristic (ROC) curve analysis.

### NGAL, Kim-1 and IL-18 detected by ELISA

Urinary NGAL, Kim-1 and IL-18 levels were analyzed using commercially available enzyme-linked immune absorbent assay (ELISA) kits: human NGAL ELISA kit (BioPorto Diagnostics, KIT036), human kidney injury molecule 1 (Kim-1) ELISA kit (Cusabio Biotech, CSB-E08807h) and human Interleukin-18 (IL-18) ELISA Kit (Invitrogen, KHC0181). All procedures were performed according to the manufacturer’s instructions.

### Urinary Hsp72 levels were assessed by ELISA

Urinary Hsp72 levels were also analyzed by ELISA, using a commercially available assay (Assay Designs ADI-EKS-715, MI, USA). Briefly, samples and standards were added to wells coated with a mouse monoclonal antibody. Hsp72 was captured by the antibody and then detected by adding a rabbit polyclonal detection antibody. The Hsp72 antibody is specific and does not react with other members of the Hsp70 family. A horseradish peroxidase conjugate binds to the detection antibody, color development was achieved by the addition of a tetramethylbenzidine substrate, and the reaction was stopped with an acid stop solution. The optical density of samples was read at 450 nm by a plate reader and was overlapped with the standard curve generated from known concentrations of recombinant Hsp72, ranging from 0.1 to 12.5 ng/ml.

### Validation test

To validate the results obtained by the ROC curves for Hsp72 in our primary phase of this study, three months after the study ended, we included a new set of patients with the same inclusion and exclusion criteria. A total of 22 patients were included, 10 with AKI and 12 with no-AKI. Because there are not previous studies evaluating Hsp72 biomarker performance for AKI in humans, we selected the cut-off value for Hsp72 according to the point in the ROC analysis at -1 day that conferred the best values for sensitivity and specificity. In addition, AUC, positive and negative predictive values were determined.

### Statistical analysis

Categorical variables are shown as frequencies and proportions, whereas continuous variables were analyzed with the Kolmogorov-Smirnov test to determine the distribution. The values of the 4 biomarkers assessed showed a normal distribution; therefore, the mean and 95% confident interval for each assessed day were calculated. Comparisons of the categorical variables were performed by the x^2^ test. To compare biomarker values between the AKI and no-AKI groups, we used the Student’s t test. ROC curves were performed to calculate biomarkers sensitivity, specificity and area under curve (AUC) 1 and 2 days before the development of AKI. For the Hsp72 validation assay, we performed a ROC analysis with the best cut-off point (1.0 ng/ml) found in the main part of the study. Statistical significance was defined when the p value was <0.05.

## Results

### The development of AKI

During the study period of 3-months, a total of 56 patients fulfilled the inclusion criteria. Seventeen were diagnosed with AKI with the AKIN criteria, which represents 30.4%, whereas, 39 (69.6%) did not develop AKI. To maintain a better proportion of positive cases, no-AKI group was composed by only 20 randomly-chosen patients.

In the 17 patients who developed AKI, AKIN I was observed in 10 (58.8%), AKIN II in 7 (41.2%) and none was in the AKIN III class. For all the AKI cases, 35.3% was diagnosed on base of a reduction in urinary output, 58.8% by serum creatinine elevation, and 5.9% by the combination of both parameters.

AKI appearance was observed in 16.2% of patients after one day of ICU admission, 5.4% after two days, and 78.4% developed AKI after three days. It is important to remember that one of the inclusion criteria was a GFR>60 ml/min.

### Main characteristics of critically ill patients with AKI and no-AKI

In Table 1, the baseline characteristics of the studied patients are listed. Thirty-seven patients were included: 17 with AKI and 20 with no-AKI. No differences were found in the mean age, gender or mortality between the patients who developed AKI compared with no-AKI patients in the ICU. The major cause of AKI was associated with septic shock, followed by cardiovascular surgery, neurological alterations, or miscellaneous causes. The main co-morbidities found were hypertension, diabetes, cancer, systemic lupus erythematous and obesity.

**Table 1:** Summary of baseline and clinical characteristics.

Variable	Total N = 37	AKI N = 17	NO AKI N = 20	*p* Value
Age (y)	51.6±20.2	54.5±22.5	49.2±18.2	0.42
Male	20 (54.1)	12 (70.6)	8 (40.0)	0.12
Mortality	15 (40.5)	8 (47.1)	7 (35.0)	0.68
**Cause of admission to ICU**				
Severe Sepsis or Septic Shock	20 (55.6)	11 (64.7)	9 (47.4)	0.38
Cardiovascular surgery	5 (13.9)	2 (11.8)	3 (15.8)	0.84
Neurological	4 (11.1)	2 (11.8)	2 (10.5)	0.72
No cardiovascular surgery	3 (8.3)	1 (5.9)	2 (10.5)	0.88
Other	4 (11.1)	1 (5.9)	3 (15.8)	0.72
**Comorbidities on admission**				
Hypertension	6 (16.2)	2 (11.8)	4 (20.0)	0.66
Diabetes	9 (24.3)	4 (23.5)	5 (25.0)	1.0
Cancer	9 (24.3)	4 (23.5)	5 (25.0)	1.0
SLE-APS	4 (10.8)	2 (11.8)	2 (10.0)	1.0
Obesity	5 (13.5)	4 (23.5)	1 (5.0)	0.15

Continuous values are represented as mean ± standard deviation; dichotomous values are *N* (%). AKI: Acute Kidney Injury. ICU: Intensive Care Unit. SLE: Systemic Lupus Erythematosus. APS: Antiphospholipid Syndrome.

### Using Kim-1, NGAL, IL-18 to establish an early diagnosis of AKI

**Figure 1:**
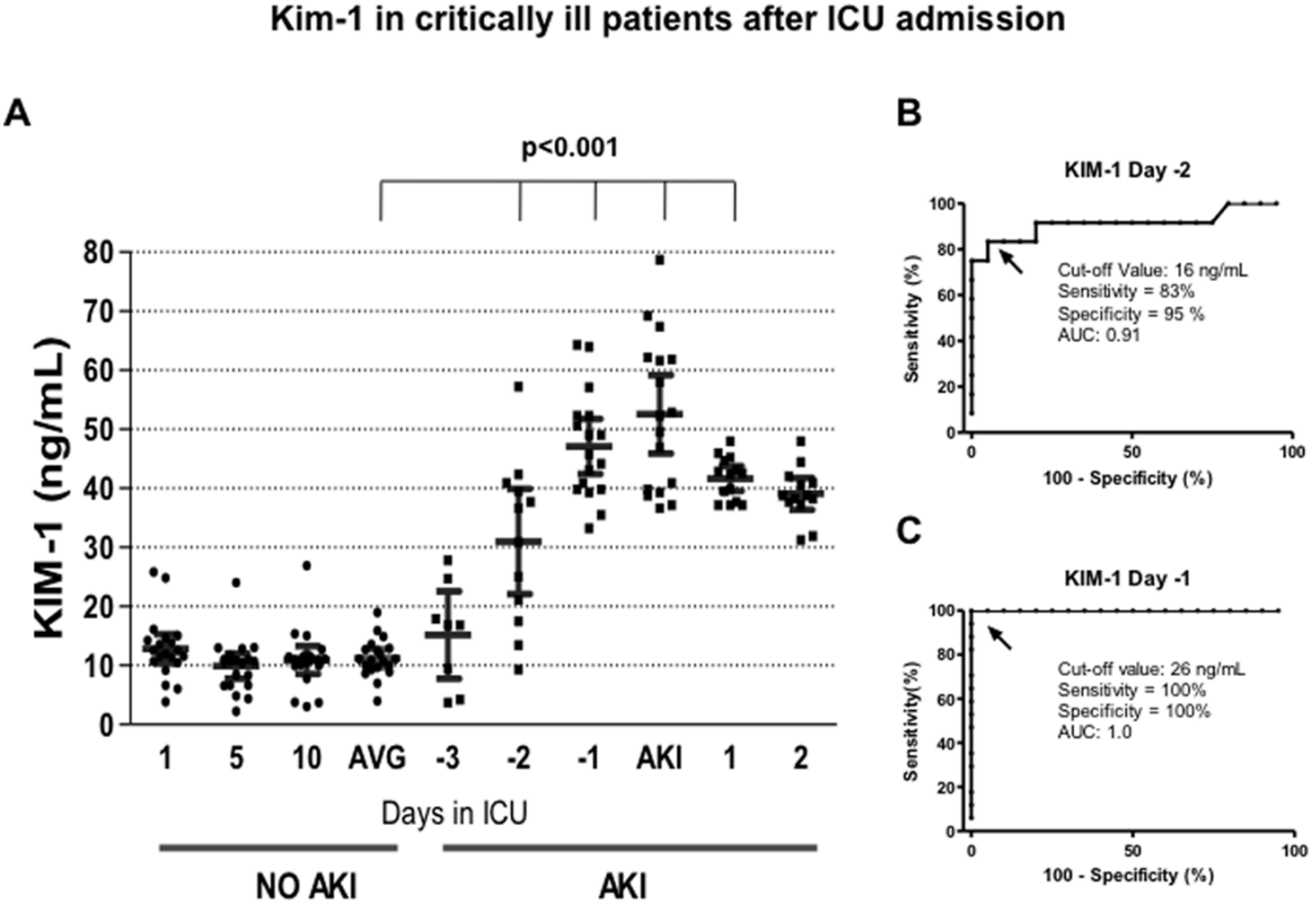
Kim-1 in critically ill patients after ICU admission. A) Urinary Kim-1 levels assessed by ELISA from AKI and no-AKI patients. In no-AKI patients, Kim-1 was measured at days 1, 5 and 10 of their stay in the ICU; whereas, in AKI patients Kim-1 was measured from ICU admission until two days after the diagnosis of AKI. Every point represents the biomarker value in each urine sample, and the lines depicted the mean and 95% confident interval. AVG = average of urinary Kim-1 levels in no-AKI patients determinated at 1, 5, and 10 days after ICU admission. B) Specificity and sensitivity of Kim-1 to detect AKI two days before the AKIN criteria, determined by ROC analysis. C) Specificity and sensitivity of Kim-1 to detect AKI one day before the AKIN criteria, determined by ROC analysis.

In critically ill patients who did not developed AKI, the biomarkers were quantified on days 1, 5 and 10 of their stay in the ICU. The media from these three measurements was compared with the media from critically ill patients who developed AKI. In the AKI population, the biomarkers were measured from 3 days before the serum creatinine elevation until 2 days after the AKI diagnosis occurred. Figure 1A shows the urinary Kim-1 levels in all patients studied. Patients with no-AKI exhibited urinary Kim-1 values approximately 10 ng/ml; this value remained unaffected throughout 10 days in the ICU. In contrast, the urinary Kim-1 levels in AKI patients rose significantly from 2 days before the AKI diagnosis using the AKIN criteria (p<0.001) and remained elevated throughout the study protocol in all the AKI patients. The AUC-ROC analysis, using a cut-off value of 16 ng/ml, revealed that at -2 day, the sensitivity and specificity of Kim-1 to detect AKI was 83 and 95%, respectively, with an AUC of 0.91 (Figure 1B). At -1 day, the sensitivity and specificity improved to 100% with an AUC of 1.00 (Figure 1C), with a cut-off value of 26 ng/ml.

**Figure 2:**
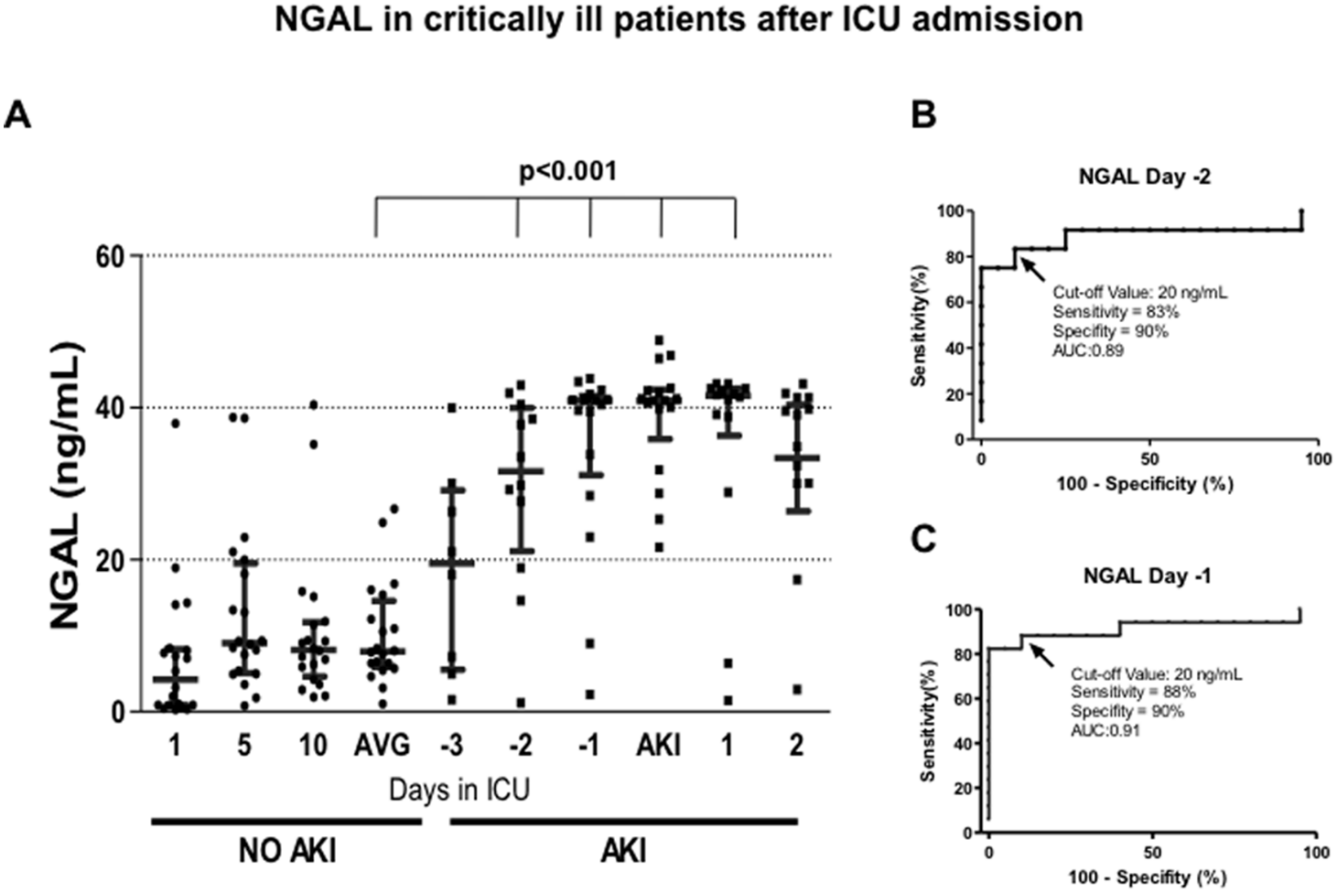
NGAL-1 in critically ill patients after ICU admission. A) Urinary NGAL levels assessed by ELISA from AKI and no-AKI patients. Every point represents the biomarker value in each urine sample, and the lines depicted the mean and 95% confident interval. AVG = average of urinary NGAL levels in no-AKI patients determinated at 1, 5, and 10 days after ICU admission B) Specificity and sensitivity of NGAL to detect AKI two days before the AKIN criteria, as determined by ROC analysis. C) Specificity and sensitivity of NGAL to detect AKI one day before the AKIN criteria, determined by ROC analysis.

Regarding NGAL, greater variability was found in the urinary NGAL levels of no-AKI patient than Kim-1 in no-AKI patients. This variability was also observed in AKI patients, but there was a significant elevation in NGAL values from -2 days (p<0.001), and similar to Kim-1, these values remained elevated (Figure 2A). Similar sensitivity and specificity of Kim-1 was observed at -2 days (83 and 90%, respectively), with an AUC of 0.89 (Figure 2B). In contrast, NGAL exhibited lesser sensitivity and specificity than Kim-1 at -1 day (88 and 90%, respectively), with an AUC of 0.91 (Figure 2C). In both cases, the same cut off value was used (20 ng/ml).

**Figure 3:**
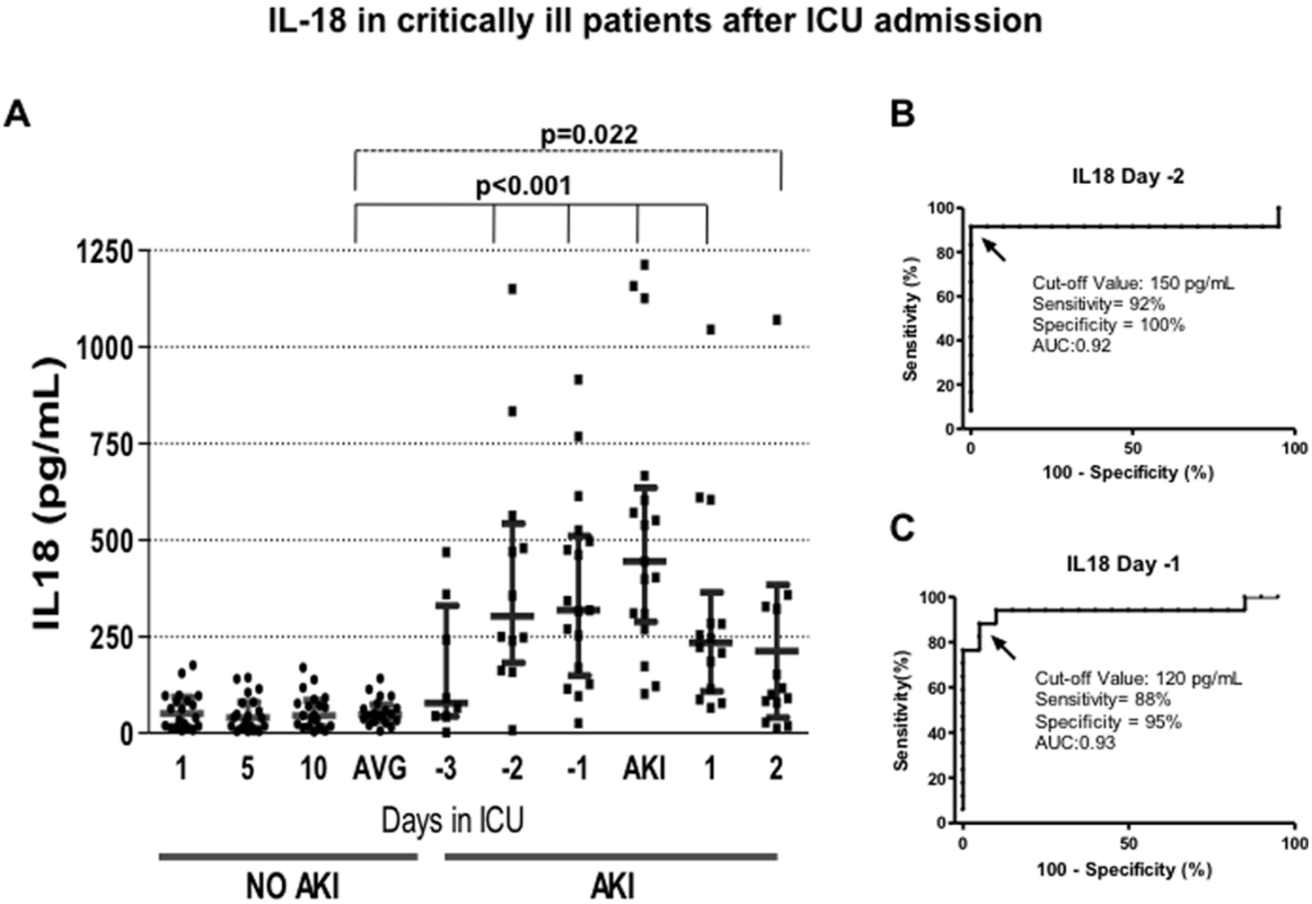
IL-18 in critically ill patients after ICU admission. A) Urinary IL-18 levels assessed by ELISA from AKI and no-AKI patients. Every point represents the biomarker value in each urine sample, and the lines depicted the mean and 95% confident interval. AVG = average of urinary IL-18 levels in no-AKI patients determinated at 1, 5, and 10 days after ICU admission. B) Specificity and sensitivity of IL-18 to detect AKI two days before the AKIN criteria, determined by ROC analysis. C) Specificity and sensitivity of IL-18 to detect AKI one day before the AKIN criteria, determined by ROC analysis.

In Figure 3A, the urinary IL-18 values are represented in both AKI and no-AKI patients during their staying in the ICU. Patients with no-AKI exhibited basal urinary IL-18 values of approximately 40 ng/ml that remained constant throughout 10 days in the ICU. In contrast, the urinary IL-18 levels rose by more than 5-fold from 2 days before the AKI diagnosis using the AKIN criteria (p<0.001). A progressive reduction in urinary IL-18 started after the AKI diagnosis; however, a great variability in each set of measurements was observed with this biomarker. The AUC-ROC analysis revealed that at -2 days, the Kim-1 sensitivity and specificity to detect AKI was 92 and 100%, respectively, with an AUC of 0.92, using a cut-off value of 150 pg/ml (Figure 3B). At -1 day, the sensitivity and specificity were 88 and 95%, respectively, with an AUC of 0.93, using a cut-off value of 120 pg/ml (Figure 3C).

**Figure 4:**
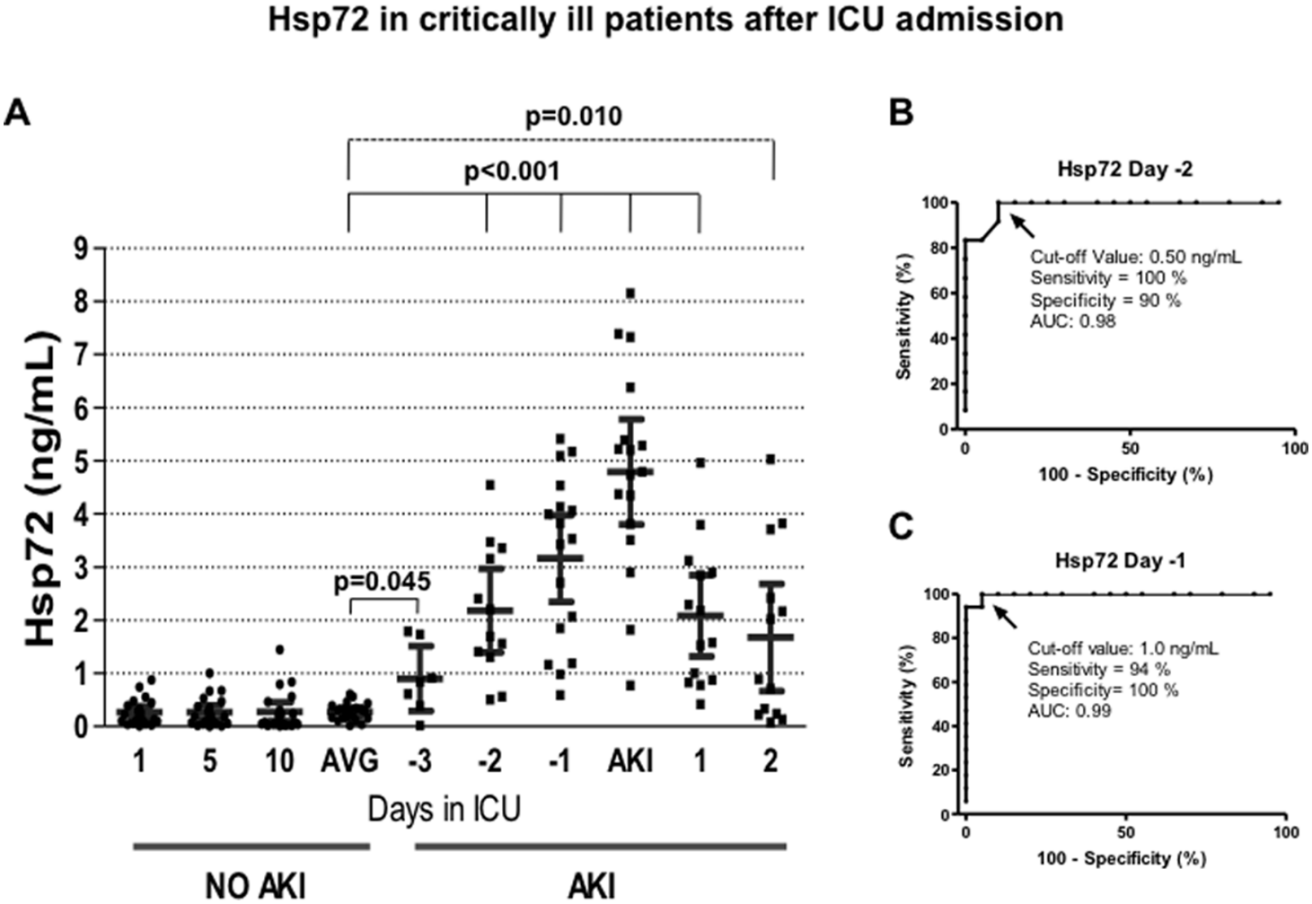
Hsp72 in critically ill patients after their admission at ICU. A) Urinary Hsp72 levels assessed by ELISA from AKI and no-AKI patients. Every point represents the biomarker value in each urine sample, and the lines depicted the mean and 95% confident interval. AVG = average of urinary Hsp72 levels in no-AKI patients determinated at 1, 5, and 10 days after ICU admission. B) Specificity and sensitivity of Hsp72 to detect AKI two days before the AKIN criteria, determined by ROC analysis. C) Specificity and sensitivity of Hsp72 to detect AKI one day before the AKIN criteria, determined by ROC analysis.

Finally, patients with no-AKI exhibited urinary Hsp72 values of approximately 0.3 ng/ml that remained unaffected throughout 10 days in the ICU. In contrast, the urinary Hsp72 levels in AKI patients rose significantly from 3 days before AKI was diagnosed with the AKIN criteria (p = 0.045). This difference was not seen with the other 3 biomarkers. A progressive increase in urinary Hsp72 levels was observed from -3 days to the diagnosis of AKI, followed by a progressive reduction until day 2 post AKI diagnosis, the last day of assessment (Figure 4A). The ROC analysis revealed that at -2 day, the sensitivity and specificity of Hsp72 to detect AKI was 100 and 90%, respectively, with an AUC of 0.98, using a cut-off value of 0.5 ng/ml (Figure 4B). At -1 day, the sensitivity and specificity was 94 and 100%, respectively with an AUC of 99%, with a cut-off value of 1.0 ng/ml (Figure 4C).

### Hsp72 validation test

For the Hsp72 validation test, a total of 22 patients were included, 10 with AKI and 12 no-AKI. The best cut-off value of Hsp72 for ROC was taken at 1 day before the AKI diagnosis. Table 2 lists the sensitivity, specificity, accuracy, and positive and negative predictive values of urinary Hsp72 in AKI and no-AKI patients. No false negatives were observed in patients with AKI and two false positives were observed in no-AKI patients. Thus, the sensibility/specificity and accuracy were 100/83.3% and 90.9%, respectively.

**Table 2:** Validation test for Hsp72 in a similar population in ICU.

		Patients withAKI	Patients withoutAKI		
	**Positive** **test**	**10**(True Positive)	**2**(False Positive)	Positive Test = **12**	**PPV = **83.3%
**Hsp72 Cut-off** **value 1.0 ng/ml**	**Negative** **Test**	**0**(False Negative)	**10**(True Negative)	Negative Test = **10**	**NPV = **100%
		**Total AKI = 10**	**Total No AKI = 12**	**All Patients = 22**	
		**Sensibility = **100%	**Specificity = **83.3%	**Accuracy = **90.9%	

Use of cut-off value one day before AKI diagnosis (AKIN criteria). PPV = Positive Predictive Value. NPV = Negative Predictive Value.

## Discussion

This is the first diagnostic test study that showed that urinary Hsp72 detection is a sensitive and early AKI biomarker for critically ill patients. Specifically, in patients who arrived in the ICU of our institution 3 days before the AKI diagnosis, Hsp72 effectively detected AKI. This performance was not seen with other assessed biomarkers. Moreover, in the urine samples at -2 and -1 days, the best detection methods of AKI were Kim-1 and Hsp72, followed by NGAL and IL-18.

The clinical impact of AKI is very clear. AKI is associated with increased morbidity, mortality, length of hospital stay, and cost. [1], [27] AKI complicates the course of disease in critically ill patients, causing several million deaths worldwide every year. Efforts to change its clinical course have failed because early detection is not possible with SCr elevation or oliguria, and novel biomarkers have not proven to be sufficiently efficacious. [28], [29] As a consequence, a delay in initiating appropriate therapies contributes to disappointing prognoses. However, a major disadvantage limiting the routine use of biomarkers is low sensitivity and specificity, which generally do not exceed 70 to 75%, for detecting early kidney damage [30]. Therefore, the creation of a kit integrating several biomarkers for the accurate diagnosis of AKI in the ICU has been proposed [28], [29].

We previously showed that Hsp72 is a reliable biomarker for the early detection of AKI in rats. Thus, urinary Hsp72 levels were adequately sensitive for stratifying different degrees of tubular injury and recovery, and for monitoring a renoprotective intervention in an experimental rat model of AKI induced by bilateral renal ischemia/reperfusion. [25] The clinical application of a new biomarker should be more accurate with earlier detectability than the current gold standard SCr. Here, we showed enough evidence indicating that Hsp72 is a specific and sensitive biomarker to detect AKI before SCr elevation is noted in critically ill patients.

Most of the previous studies using biomarkers have focused on and been validated in situations in which the time of renal injury can be easily known, such as during cardiac surgery, administration of intravenous contrast, or other nephrotoxic agents. [24], [31], [32]–[38] In contrast, this study was designed to focus on critically ill patients, who clinicians see in their daily practice, and there is uncertainty about the precise moment in which AKI starts. Here, we found that the increase in urinary Hsp72 levels preceded the rise in creatinine and reduction in urinary volume by up to 3 days.

Although, most of the other biomarkers assessed showed similar ability to predict AKI 24-h before the AKIN criteria were fulfilled, Hsp72 was clearly superior because it was the earliest detectable AKI biomarker and was extremely sensitive and specific.

In this study, we found that most of the cases of AKI were classified as AKIN 1, a class seen by clinicians as having little relevance or clinical impact. However, in the context of the ICU, early detection of mild cases of AKI will improve renal perfusion and can modify the natural history of AKI, with the potential to reduce the frequency of AKI and CKD development.

Although, there is uncertainty on how and when measurements of any novel biomarker should be completed because in clinical practice, this is a challenge. [39] Most critically ill patients developed AKI in the first three days after admission. Thus, to efficiently detect AKI before conventional markers, the balance between cost and benefit would favor daily urinary Hsp72 detection, from ICU admission to three to five days after ICU discharge.

To reinforce our results, we validated with a different ICU patient population and obtained similar results. The strength of our study is based in that all included patients had the same critical conditions and none healthy subject was included, thus Hsp72 elevation was not related to the clinical condition.

The ability of urinary Hsp72 levels to detect AKI could differ depending on the cause of AKI, such as contrast-induced nephropathy or post-surgical AKI. Thus, more studies are necessary to prove the applicability of Hsp72 in these entities.

The limitations of our study are the number of critically ill patients included and the exclusion of patients with pre-existing chronic kidney disease. Thus the applicability of this biomarker in this population of patients have to wait until the validation will be performed.

Our data show that Hsp72 is a sensitive biomarker for detecting early AKI in critically ill patients without pre-existing chronic kidney disease, which could be helpful to establish early interventions intended to prevent or reduce AKI severity and improve prognosis.
